# Ceftriaxone-Induced Encephalopathy in a Patient With Chronic Kidney Disease

**DOI:** 10.7759/cureus.54476

**Published:** 2024-02-19

**Authors:** Ana Filipa Martins, Mónica Dias, Rita Matos Sousa, Maria João Regadas

**Affiliations:** 1 Medicina Interna, Hospital de Braga, Braga, PRT; 2 Cardiology, Hospital de Braga, Braga, PRT; 3 Clinical Sciences, Escola de Medicina Universidade do Minho, Braga, PRT; 4 Internal Medicine, Hospital de Braga, Braga, PRT

**Keywords:** central nervous system disorders, chronic kidney disease, ceftriaxone, cephalosporins, neurotoxicity, drug-induced encephalopathy

## Abstract

Neurotoxicity is an acknowledged side effect of third and fourth-generation cephalosporins, but its occurrence with ceftriaxone is not widely recognized. This article presents a case involving a 56-year-old woman with multiple comorbidities who sought medical attention after experiencing lipothymia. The initial diagnosis suggested a urinary tract infection with acute kidney failure, leading to the initiation of ceftriaxone and hemodialysis. Subsequently, the patient exhibited a progressive deterioration of her neurological state, characterized by agitation and chorea. Metabolic encephalopathy, seizure/nonconvulsive status epilepticus, and acute central nervous system lesions were considered primary differential diagnoses, all of which were subsequently ruled out through thorough investigations. Days later, a remarkable recovery of the patient's neurological state was observed. A retrospective analysis revealed a correlation between the improvement and the fourth day of antimicrobial suspension. Consequently, a presumptive diagnosis of ceftriaxone-induced encephalopathy was made. This unusual case underscores the importance of recognizing the potential for pharmacological encephalopathy, particularly with ceftriaxone, and emphasizes its reversibility upon discontinuation of the implicated drug. Clinicians should remain vigilant to this uncommon adverse effect, promoting timely intervention and improved patient outcomes.

## Introduction

While neurotoxicity, including encephalopathy, has been documented as a potential side effect of third and fourth-generation cephalosporins, its association with ceftriaxone remains relatively uncommon and challenging to diagnose, supported by limited case reports in the literature [1-3]. The European Medicines Agency (EMA) recognized this underreported phenomenon in 2020, recommending the inclusion of encephalopathy as a potential side effect of ceftriaxone, particularly in elderly patients with severe renal impairment or central nervous system disorders [4]. This recommendation gains significance given the frequent utilization of ceftriaxone in the treatment of urinary and respiratory infections in individuals with compromised kidney function, obviating the need for dosage adjustments.

Here, we present a compelling case involving a 56-year-old woman burdened with multiple comorbidities, including cerebrovascular disease and chronic kidney disease. She presented with acute reversible encephalopathy after being treated for a urinary infection with ceftriaxone. This article provides a detailed account of the case's progression and the diagnostic intricacies encountered during the patient's stay in the Internal Medicine ward.

The significance of this case was acknowledged by its presentation as a poster at the 20th European Congress of Internal Medicine held from June 9-11, 2022.

## Case presentation

A 56-year-old woman sought medical attention in the emergency department following an episode of lipothymia. Her extensive medical history included stage V chronic kidney disease (CKD), arterial hypertension, type II diabetes mellitus, morbid obesity, ischemic cardiomyopathy with preserved ejection fraction, ischemic stroke, and iron deficiency anemia. The patient adhered to a medication regimen comprising acetylsalicylic acid (100 mg/day), bisoprolol (2.5 mg/day), atorvastatin (80 mg/day), dapagliflozin (10 mg/day), linagliptin (5 mg/day), amlodipine (10 mg/day), calcium carbonate (1000 mg/day), and colecalciferol (800 IU/day).

Upon arrival at the emergency room, the patient exhibited hemodynamic stability, was apyretic, and was described as fully conscious, cooperative, and oriented. Her speech was coherent and easily understood, and she followed instructions appropriately. Her pupils were isochoric and isoreactive. Furthermore, there were no abnormalities detected in the assessment of cranial nerves, and the patient showed no deficits in strength or sensation in all four limbs. However, blood analysis revealed a worsening of anemia (Hb 6.1 g/dL - N12 to 16g/dL) and elevated inflammatory parameters, including leukocytosis (leukocytes 15,550/µL - N4,000 to 11,000/µL) and C-reactive protein (246.7 mg/L - N<0.5mg/L). Kidney function was further compromised, with a serum creatinine of 14.43mg/dL (baseline 4.9mg/dL - N0.6 to 1.1 mg/dL) and serum urea reaching 274 mg/dL (N20 to 50mg/dL). Additional findings included hyperkalemia (K+ 5.3 mmol/L - N3.6 to 5.2mmol/L), metabolic acidosis (pH 7.01 - N7.35 to 7.45; pCO2 16 mmHg - N35 to 45mmHg; HCO3- 4.5 mmol/L - N22 to 26mEq/L), and ketonemia (2.7 mmol/L - N<0.6mmol/L). Urinalysis revealed leukocyturia and ketonuria (5 mg/dL - N<5mg/dL). Renal ultrasound indicated chronic nephropathy without signs of urinary tract obstruction.

Based on these findings, a diagnosis of acute urinary infection with concurrent acute kidney failure and euglycemic diabetic ketoacidosis, and exacerbated anemia was established. Treatment was started with ceftriaxone 2 g, intravenous fluids, bicarbonate, and intravenous insulin. The patient received a transfusion of two units of red blood cells and was subsequently transferred to the intermediate care unit of our central hospital for hemodialysis (HD). Following two days, the patient was admitted to the Internal Medicine ward.

Despite medical intervention, the patient's mental state progressively deteriorated, exhibiting confusion, disorientation, and an inability to engage in coherent conversation. Considering the presence of a bladder catheter, along with ongoing non-specific systemic complaints and neurological deterioration, the decision was made to extend the antibiotic course. This extension was prompted by the potential impact of a urinary tract infection on the patient's current clinical status. On the 10th day of hospitalization and ceftriaxone treatment, apathy interspersed with brief periods of agitation and chorea were observed. No focal neurological signs or convulsive movements manifested.

Given the patient's medical history, the primary consideration was a metabolic cause, specifically uremic encephalopathy. To explore this possibility, additional HD sessions were conducted at short intervals. Despite these efforts, there were only marginal improvements in neurological conditions and a slight decrease in serum urea values. While clinical presentation and certain laboratory findings hinted at uremic encephalopathy, the lack of significant improvement post-HD sessions made this diagnosis less likely, favoring alternative etiologies. Various metabolic disturbances, such as ionic and electrolyte imbalances, metabolic acidosis, hypoglycemia or hyperglycemia, and acute hepatic failure, were considered as alternative explanations, but all were found to be normal or were corrected through early HD sessions. The patient did not exhibit hypoxia, acute signs of anemia, or hypotension that could justify cerebral hypoxia.

**Figure 1:**
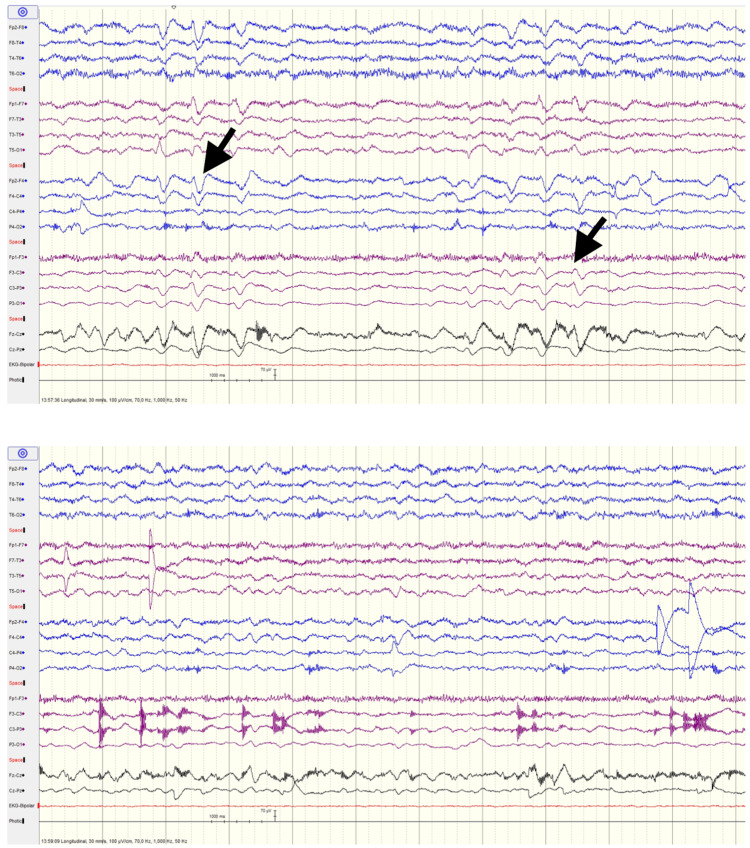
EEG carried out at the pinnacle of the patient's most evident cognitive deterioration This EEG was conducted on a restless patient in both wakefulness and drowsiness, incorporating intermittent photic stimulation (IPS). The patient exhibited limited cooperation during the examination, with numerous movements observed throughout the study. The recording showed numerous artifacts, with a baseline activity at 5 Hz, posterior, symmetric, reactive, irregular, and of low amplitude. The slow activity was inscribed with variable localization (arrows). No clear recording of spikes and/or sharp waves, or other focal or generalized paroxysms, was observed. No electroclinical seizures were observed. In conclusion, it shows moderate encephalopathy with inscribed slow activity of variable localization, without clear evidence of epileptiform activity. The recording is overlaid with numerous artifacts.

At the peak of the most pronounced decline in mental status, despite the absence of epileptic crisis, nystagmoid eye movement, or automatisms, the possibility of epilepsy or nonconvulsive status epilepticus was contemplated. An EEG was performed and revealed moderate encephalopathy with slow activity inscription and variable location, devoid of evident epileptiform activity (Figure 1). Head CT scans showed signs of ischemic leukoencephalopathy, a sequelae injury from a prior ischemic event, and multiple injuries in the basal ganglia suggestive of sequels of lacunar strokes. Furthermore, the CT scan indicated a higher degree of brain atrophy than expected for the patient's age. Neurology specialists observed the patient without finding evidence of a neurological disease explaining the clinical findings. 

At this time, an additional complication surfaced, the patient developed Clostridium infection and started 500 mg of oral vancomycin daily for 10 days, resulting in complete symptom resolution by the fifth day of treatment.

Notably, on day 17, mental improvements were discerned, culminating in a full recovery of the initial neurological condition. Revisiting the timeline of events, it was observed that the patient's mental status improved four days after the suspension of ceftriaxone, with no other changes in medication or clinical decisions. Consequently, by exclusion, the diagnosis of ceftriaxone-induced encephalopathy was established. A follow-up EEG, conducted twelve days after ceftriaxone suspension, was unremarkable. Upon discharge, she demonstrated consciousness, orientation, an inability to recall the preceding days, conversational ability, and full fine motor skills capacity.

## Discussion

Ceftriaxone, a widely prescribed third-generation cephalosporin, is favored for its broad-spectrum antimicrobial activity against Gram-positive and Gram-negative bacteria and unique pharmacokinetic properties [5]. While generally well-tolerated, ceftriaxone is associated with potential side effects, including infrequent but reported neurotoxicity, manifesting as myoclonus, seizures, and encephalopathy/altered mental status [1,2,6-9]. The exact pathophysiology is not totally elucidated, but it is thought to involve decreased γ-aminobutyric acid (GABA) release, leading to increased excitatory neurotransmission [10,11].

Ceftriaxone is predominantly excreted via the urinary route, with the remaining portion eliminated through biliary secretion. The reduction in renal clearance associated with kidney disease or aging is typically counterbalanced by an augmented elimination through the fecal route. Concurrently, ceftriaxone demonstrates the capacity to maintain its concentration even in the presence of renal impairment. This phenomenon elucidates the rationale for the lack of dosage adjustment requirements based on renal function [12].

Contrarily, the literature suggests heightened susceptibility to ceftriaxone neurotoxicity in renal patients. The neurotoxicity correlates with serum concentration rather than the dosage used, with a lack of linear correlation between creatinine clearance and plasma clearance of ceftriaxone [12,13]. In a small percentage of patients with end-stage renal disease patients on HD, the elimination half-life is notably prolonged, and ceftriaxone is not effectively removed during HD, explaining the persistence of neurological symptoms post-treatment sessions [13,14].

Predisposing factors for ceftriaxone-induced neurotoxicity include renal insufficiency and pre-existing central nervous system abnormalities. Our patient, displaying renal impairment and a history of ischemic brain disease as evidenced by CT scans, aligned with these risk factors. EEG patterns associated with cephalosporin neurotoxicity include diffuse slow-wave delta activity, semi-periodic triphasic sharp wave activity, or frank periodic discharges, often localized to frontal regions [11]. These findings, some of them present in our patient’s EEG, may also be present in patients with other metabolic encephalopathies.

The central nervous system adverse effects of ceftriaxone are likely underdiagnosed and underreported, potentially due to their underrecognition and limited documentation in the literature. Pharmacovigilance reports highlight these effects more prominently than published studies. Consequently, heightened clinical vigilance is essential, particularly when patients present with neurological impairment during ceftriaxone use. Discontinuation of the drug may lead to significant neurological improvement. Previous studies were able to correlate not only plasma, but also cerebrospinal fluid concentration of ceftriaxone with neurotoxicity [3,6,9]. Regrettably, an assessment of these concentrations was unattainable, prompting a presumptive diagnosis derived from the temporal progression of the patient's clinical course.

While a literature search revealed some cases akin to ours, reports on ceftriaxone-induced encephalopathy remain scarce, indicating the imperative for further investigation. Cases with diverse underlying conditions, ranging from enteric fever to endocarditis, pneumonia, diverticulitis, or pyogenic arthritis, propose that this phenomenon is not exclusive to urinary tract infection patients [1,3,15,16]. Published cases commonly describe altered mental states without apparent causes, with initial differential diagnoses considering metabolic encephalopathies, progressively ruled out, as evidenced in this clinical case. Patients consistently exhibited unremarkable head CT scans and variable EEG changes, raising suspicion of ceftriaxone neurotoxicity only after drug suspension and subsequent mental state improvement. The association with acute renal failure, frequently linked to severe chronic renal disease and cerebrovascular/coronary heart disease, further accentuates the necessity for recognizing this adverse effect [1-3,6,15].

Moreover, it is essential to underscore the lack of correlation between the acute worsening of mental status and Clostridium infection or the use of vancomycin, dispelling potential confounding factors in our patient's case. The neurological symptoms preceded the onset of diarrhea, reinforcing the likelihood of ceftriaxone-induced encephalopathy as the primary causative factor.

## Conclusions

This rare case aims to raise awareness among physicians regarding the potential occurrence of pharmacological encephalopathy, particularly in vulnerable patients. A heightened clinical suspicion is imperative for diagnosing ceftriaxone-induced encephalopathy, recognizing it as a reversible cause of acute encephalopathy. Future investigations could explore the feasibility of establishing ceftriaxone dose adjustments based on renal function and/or monitoring its plasma concentrations. This consideration becomes even more crucial in elderly patients (>65 years) and/or those with renal impairment, given their elevated risk of experiencing serious central nervous system adverse effects.
